# An automated data extraction and classification pipeline to identify a novel type of neuron within the dorsal striatum based on single-cell patch clamp and confocal imaging data

**DOI:** 10.1016/j.dib.2020.106148

**Published:** 2020-08-07

**Authors:** Miaomiao Mao, Aditya Nair, George J. Augustine

**Affiliations:** a ^a^Lee Kong Chian School of Medicine, Nanyang Technological University, Singapore, Singapore; b ^b^Institute of Molecular and Cell Biology, Singapore, Singapore; c ^c^Singapore Bioimaging Consortium, Agency of Science and Technology, Singapore

**Keywords:** Neuron classification, Whole-cell patch clamping, Two-photon imaging, Intrinsic electrical properties, Unsupervised hierarchical clustering, Machine-learning aided classification

## Abstract

We employed electrophysiological and fluorescence imaging techniques to describe the characteristics of a novel type of neuron discovered in the mouse dorsal striatum. Transgenic mice that express YFP-tagged channelrhodopsin-2 (ChR2) in neurons driven by the promoter for tyrosine hydroxylase (TH) were used and the intrinsic electrical properties of YFP-positive neurons in the dorsal striatum of these mice were characterized using whole-cell patch clamping in acute brain slices. Passive membrane properties - such as membrane capacitance, resting membrane potential and input resistance -and action potential properties- such as amplitude, kinetics and adaptation - were extracted from raw data files. Filling these neurons with neurobiotin enabled visualization of neuronal morphology via immunohistochemical labeling with streptavidin-conjugated fluorophore. Subsequent two-photon imaging allowed analyses of morphological properties such as somaticsize, dendritic branching (Sholl analysis) and density of dendritic spines. Unbiased analyses and hierarchical clustering of both morphological and functional data allowed us to identify a previously undescribed type of striatal neuron with unique properties. To facilitate identification of this new cell type, an end-to-end automated electrophysiology pipeline was developed that extracts relevant parameters and determines striatal neuron identity using neural-network based classifiers. These data and the software tool will permit other investigators to identify this novel type of neuron in their studiesand thereby better understand theroles thatthese neuronsplay in dorsal striatum circuitry.

**Specifications Table**

**Table:** 

**Subject**	Neuroscience (General)
**Specific subject area**	Neural circuitry: the study of how different types of neurons are wired and communicate with each other.
**Type of data**	TableImageGraphFigure
**How data were acquired**	Electrophysiological data were acquired using whole-cell patch clamping in mouse acute brain slices. Brain slices were sectioned using a vibrating microtome (Leica VT1200S). Patch clamping data were acquired with a Multiclamp 700B amplifier and a Digidata 1330a interface with pClamp 10.4 software (all from Molecular Devices).Morphological data were acquired using a two-photon microscope (Olympus FV-1000).
**Data format**	Raw:.abf (pClamp data).tif (Olympus 2P imaging data)Analyzed
**Parameters for data collection**	As the cell's condition affects the quality of electrophysiological recording and reliability of data, recordings with series resistance greater than 25 MΩ, resting membrane potential (RMP) more depolarized than −50 mV or fluctuations greater than 10% were excluded from analysis.
**Description of data collection**	Tyrosine hydroxylase (TH)-Cre transgenic mice were mated with Ai32 mice expressing YFP-tagged channelrhodopsin 2 (ChR2) behind a floxed stop cassette to generate mice expressing ChR2-YFP in TH—Cre expressing neurons. Parasagittal slices of 300 µm thickness, containing the dorsal striatum, were cut with a vibratome. Cells within the dorsal striatum expressing ChR2-YFP were visualized using a two-photon microscope (Olympus FV-1000) and somatic whole-cell patch clamp recordings were performed using a MultiClamp 700B amplifier and Digidata 1330a (Molecular Devices).Blue light (458 nm) was used to photostimulate neurons expressing ChR2.Neurobiotin was diffused into cells via the patch pipette. Brain slices were then fixed in 4% paraformaldehyde and incubated in streptavidin-Alexa 633. Neurobiotin-streptavidin within cells was imaged via a two-photon microscope (Olympus FV-1000). Z-stack images were obtained for each labelled cell and cellular morphology was digitally reconstructed using FIJI/ImageJ.
**Data source location**	Institution: Nanyang Technological UniversityCity/Town/Region: SingaporeCountry: Singapore
**Data accessibility**	On public repository.Repository name: Mendeley DataData identification number:doi:10.17632/sm48fzszpt.2doi:10.17632/j5h8kvh4dj.1doi: 10.17632/nvzcjvws2j.1Direct URL to electrophysiology data:https://data.mendeley.com/datasets/sm48fzszpt/2Direct URL to imaging data:In Fig 9 of Mao et al., 2019:https://data.mendeley.com/datasets/j5h8kvh4dj/1In Fig 12 of Mao et al., 2019https://data.mendeley.com/datasets/nvzcjvws2j/1Direct URL to software created for analysis: https://striatum.shinyapps.io/fans/
**Related research article**	Miaomiao Mao, Aditya Nair and George J. Augustine. A Novel Type of Neuron Within the Dorsal Striatum. Frontiers in Neural Circuits. doi: 10.3389/fncir.2019.00032

## 1 Value of the data

• These data present in detail the basis for identifying a novel type of neuron in the dorsal striatum that has unique intrinsic electrical properties and morphological properties; future studies can use these data as a reference.

The software tool that we provide can help researchers identify the novel neuron in their future studies.

• Researchers who study striatal circuitry can benefit from these data; in particular, the distinct properties of the novel neuron type lend its unique computational properties that can be examined in modeling studies that utilize our dataset.

## 2 Data description

• All .abf files are electrophysiology data recorded using pClamp 10.4 (Molecular Devices). They are current clamp recordings from YFP^+^cells in the dorsal striatum; stimulating currents ranged from −200 to +200 pA, in 50 pA increments. Each current pulse lasted one second.These recordings were used to extract and analyze input resistance, sag amplitude andAP properties (e.g. peak amplitude, half-width, afterhyperpolarization and adaptations).
• Despite being a proprietary format, files in the .abf format can be analysed using the free software Clampfit provided by Molecular devices available here: https://bit.ly/3etT5tZ. These data can also be converted into the open source format Neurodata Without Borders (NWB) and analysed using software provided by the Allen Institute for Brain Science here: https://bit.ly/2Yr906K
• In addition to data, we have created a freely available tool that allows users to extract electrophysiology data from .abf files in an automated and reproducible manner. A classifier is then used to determine cell identity from the extracted data. All tif. files are two-photon imaging data acquired using an Olympus FV-1000 microscope with a 25x objective (NA=1.05).All imaging dataare Z-stack images.
• Analysed data can be found in the figures in Mao et al., 2019 (doi: 10.3389/fncir.2019.00032).

## 3 Experimental design, materials, and methods

As the data collection process has already been described in the table above, here we will focus on the data analyses.

### 3.1 Electrophysiological classification of neuron types

A total of 12 intrinsic electrical properties were used to classify ChR2-YFP expressing neurons of the dorsal striatum; these properties were clustered using unsupervised hierarchical clustering (see Table 1 in Mao et al., 2019) [4]. These includemembrane capacitance, input resistance, resting membrane potential, voltage sag during hyperpolarization, threshold current for evoking an AP (rheobase), AP amplitude, after-hyperpolarization (AHP) amplitude, duration (half-width), and maximum rise and fall rates of APs. In addition, AP frequency and amplitude adaptation patterns were determined from voltage responses to the smallest current step that elicited at least 10 APs. All properties were extracted from raw data using custom scripts written in Matlab (Mathworks).

To cluster the data, we used PCA with singular value decomposition to reduce the dimensionality of our electrophysiology dataset. This was implemented using the pcaMethods package in R. For unsupervised hierarchical clustering, we used the three principle components that accounted for 69% of the total variance in our dataset. We identified the optimal cluster number using silhouette analysis implemented in R and visualized clusters using a dendrogram using the R package Clustvis.

### 3.2 Morphological analysis and classification of neuron types

For morphological analysis of neurobiotin-labeled neurons, cells were digitally reconstructed using a software suite for reconstruction of neuronal morphology (AdReconstructor). Firstly, pre-processing algorithms were applied to remove common sources of noise, such as non-specific background staining and neurobiotin leakage. Reconstruction of cell morphology was performed using the All Path Pruning algorithms developed by Xiao and Peng (2013) and post-processing measures were applied to ensure reconstructionaccuracy [1]. The automated traces obtained in the SWC format (open source) were subsequently quantified using l-Measure to obtain parameters such as surface area against path distance and spine density. Dendritic branching was analyzedby Sholl analysis utilizing the Simple Neurite Tracer plugin in ImageJ.

To examine the relationship between electrophysiological and morphological properties, we also performed unsupervised hierarchical clustering of morphological features extracted from 15 digitally reconstructed neurons filled with neurobiotin. The morphological properties extracted were based on definitions used in Scorcioni et al. (2008) [2] and Gouwens et al. (2018) [3], which are also described in detail in our related publication (See Table 2 in Mao et al., 2019) [4]. We have extracted a total of 23 properties using the ‘‘compute global feature’’ plug-in in Vaa3D. Dimensionality reduction was performed using PC analysis, as described for electrophysiological classifications.

### 3.3 Striatal TH-Interneuron classifier: an automated extraction and classification tool fordetermining striatal th interneuron identity

To allow users to interact with the data presented in this paper, we have created a freely available tool that performs automated extraction of electrophysiological parameters used to distinguish striatal TH-interneuron identity (See Table 2 in Mao et al., 2019) [4].

The program is written in the R programming language [5], using the Shiny package to create a graphical user interface. Electrophysiological parameters are extracted using custom scripts using definitions used in Mao et al., 2019. A feedforward neural network with a single hidden layer consisting of 13 neurons was trained to predict cell identities of 3 classes: medium spiny neurons, THINs and FANs with 10-fold cross validation. The network obtained an accuracy of 91.3% on held out test data. To interpret the classification from the trained network, we used the LIME package in R to obtain a list of features and weights that contributed most to a given classification [6]. The classifier is provided as an editable table where users may paste electrophysiological parameters extracted either through our automated pipeline or by any other means.

## Declaration of Competing Interest

The authors declare that they have no known competing financial interests or personal relationships which have, or could be perceived to have influenced the work reported in this article.

## Appendix Supplementary materials

Image, application 1

## References

[1] Xiao H., Peng H. (2013). APP2: automatic tracing of 3D neuron morphology based on hierarchical pruning of a gray-weighted image distance-tree. Bioinformatics.

[2] Scorcioni R., Polavaram S., Ascoli G.A. (2008). l-Measure: a web-accessible tool for the analysis, comparison and search of digital reconstructions of neuronal morphologies. Nat. Protoc.

[3] Gouwens N.W., Sorensen S.A., Berg J., Lee C., Jarsky T., Ting J., Sunkin S.M., Feng D., Anastassiou C.A., Barkan E., Bickley K., Blesie N., Braun T., Brouner K., Budzillo A., Caldejon S., Casper T., Castelli D., Chong P., Crichton K., Cuhaciyan C., Daigle T.L., Dalley R., Dee N., Desta T., Ding S.-.L., Dingman S., Doperalski A., Dotson N., Egdorf T., Fisher M., de Frates R.A., Garren E., Garwood M., Gary A., Gaudreault N., Godfrey K., Gorham M., Gu H., Habel C., Hadley K., Harrington J., Harris J.A., Henry A., Hill D., Josephsen S., Kebede S., Kim L., Kroll M., Lee B., Lemon T., Link K.E., Liu X., Long B., Mann R., McGraw M., Mihalas S., Mukora A., Murphy G.J., Ng L., Ngo K., Nguyen T.N., Nicovich P.R., Oldre A., Park D., Parry S., Perkins J., Potekhina L., Reid D., Robertson M., Sandman D., Schroedter M., Slaughterbeck C., Soler-Llavina G., Sulc J., Szafer A., Tasic B., Taskin N., Teeter C., Thatra N., Tung H., Wakeman W., Williams G., Young R., Zhou Z., Farrell C., Peng H., Hawrylycz M.J., Lein E., Ng L., Arkhipov A., Bernard A., Phillips J.W., Zeng H., Koch C. (2019). Classification of electrophysiological and morphological neuron types in the mouse visual cortex. Nat. Neurosci.

[4] Mao M., Nair A., Augustine G.J. (2019). A Novel Type of Neuron Within the Dorsal Striatum. Front. Neural Circuits.

[5] R.C. Team (2017). R: A language and Environment For Statistical Computing.

[6] Ribeiro M.T., Singh S., Guestrin C. (2016). Why Should I Trust You?. Proc. 22nd ACM SIGKDD Int. Conf. Knowl. Discov. Data Min. - KDD ’16.

